# Orthostatic Hypotension and the Risk of Congestive Heart Failure: A Meta-Analysis of Prospective Cohort Studies

**DOI:** 10.1371/journal.pone.0063169

**Published:** 2013-05-13

**Authors:** Wei Xin, Zhiqin Lin, Xiaoying Li

**Affiliations:** 1 First Department of Geriatric Cardiovascular Medicine, Chinese PLA General Hospital, Beijing, China; 2 Department of Cardiovascular Medicine, Affiliated Hospital of Guiyang Medical College, Guizhou, China; Temple University, United States of America

## Abstract

**Background:**

Orthostatic hypotension (OH) has been related to the increased risk of future congestive heart failure (CHF) events. However, the overall quantitative estimate of predictive ability of OH for CHF has not been determined. We therefore performed a meta-analysis to investigate the association between OH and incident CHF.

**Methods:**

Prospective cohort studies relevant to the aim of the study were identified by searching of Medline and Embase databases up to December 25, 2012 without restrictions and by reviewing the reference lists from retrieved articles.

**Results:**

A total of 51270 subjects and 3603 incident CHF cases from 4 prospective cohorts were included in the meta-analysis. Using random effect model, the pooled result indicated that presence of OH at baseline was significantly associated with an increased risk for future CHF outcomes (adjusted hazard ratio: 1.30, 95% confidence interval 1.09–1.55; p = 0.004). Results of stratified analysis suggested that the association between OH and CHF incidence seemed to be significant in middle-age subjects, or the individuals with hypertension and diabetes at baseline, but did not significant in the elderly subjects or those without hypertension or diabetes.

**Conclusions:**

Our meta-analysis confirmed that presence of OH is related to a significant increased risk for development of CHF in the future. Studies are needed to explore the potential mechanisms underlying this association. More importantly, screen for OH may be of great clinical significance for the early identification of subjects at higher risk for development of CHF.

## Introduction

Despite impressive advances in medical management and device therapy in recent decades, congestive heart failure (CHF) remains a major and growing public health problem that is associated with frequent hospitalization, poor quality of life and high rates of morbidity and mortality [1], [2]. Aggressive modification of known CHF risk factors is critical for the primary prevention of CHF. However, although some common risk factors, including diabetes mellitus (DM), hypertension and coronary artery disease (CAD) et al. [3]–[5], have been explored in previous population-based studies, early identity of individuals who have higher risk for developing CHF is still difficult, especially for the general population without apparent cardiovascular diseases (CVD).

Orthostatic hypotension (OH), described as abnormal fall in blood pressure (BP) on standing, has been observed to be prevalent in both the elderly and the middle-age population [6]. Although individuals with OH are often asymptomatic, evidence from several epidemiologic studies indicated that these subjects are associated with increased risk of future CVD or mortality [7]–[9]. Recently, some prospective cohort studies have found a significant association between OH and risk of incident CHF [10], [11], while the findings have not always been consistent [12], [13]. Therefore, we performed a meta-analysis to provide an overview of relevant studies and to provide an overall quantitative estimate of predictive ability of OH for CHF. Moreover, we tried to clarify which subgroups of subjects with OH are at higher risk for incident CHF.

## Methods

We followed the Meta-Analysis of Observational Studies in Epidemiology protocol [14] and Cochrane Handbook guidelines [15] throughout the design, implementation, analysis, and reporting for this study.

### Literature Searching

Pubmed and Embase databases were searched for relevant records, using the terms “orthostatic hypotension”, “postural hypotension” in combination with “heart failure”, “cardiac dysfunction” and “cardiomyopathy”. The search was limited to studies in humans without restriction of languages. We also analyzed reference lists of original and review articles using a manual approach. The final literature search was performed on December 25^th^, 2012.

### Study Selection

Studies were included for analysis if they met the following criteria: 1) published as full-length article or abstract in any language; 2) reported as prospective cohort studies in humans (regardless of sample size and follow-up duration); 3) included adult population (≥18 years of age) without prevalent CHF at baseline; 4) OH was identified at baseline, and defined as a decline in systolic BP≥20 mmHg or a decline in diastolic BP≥10 mmHg within three minutes from supine to standing according to the international consensus criteria [16]; 5) documented the CHF outcome on follow-up, and reported the multivariable-adjusted relative risk (RR) or hazard ratio (HR), and their corresponding 95% confidence intervals (CI) for CHF incidence comparing individuals with OH at baseline to those without OH.

### Data Extraction and Quality Assessment

Two authors (WX and ZL) independently performed the literature searching, data extraction, and quality assessment according to the inclusion criteria. Discrepancies were resolved by consensus. Extracted data include: 1) general information: year of publication and country where the study was conducted; 2) baseline characteristics of the study population: source of the population, numbers of the participants included, age, gender, mean baseline body mass index (BMI), proportions of participants with hypertension, DM and coronary artery disease (CAD); 3) definition and prevalence of OH and at baseline; 4) definition of CHF outcome; 5) follow-up data: follow-up duration, numbers of CHF cases, and variables adjusted for analysis; 6) outcome data: association of OH and risk of CHF reported as HRs and 95% CIs.

The quality of each study was assessed using the Newcastle-Ottawa Scale [17]. This scale ranges from 1 to 9 stars and judges each study on three broad categories: selection of the study groups; the comparability of the groups; and the ascertainment of the outcome of interest.

### Statistical Analysis and Data Synthesis

RevMan (Version 5.1; Cochrane Collaboration, Oxford, UK) and STATA software (Version 12.0; Stata Corporation, College Station, TX) were used for the meta-analysis. We transformed hazard ratios by taking their natural logarithms and calculating standard errors from the corresponding 95% CI as follows: (Ln[upper limit of CI] - Ln[lower limit of CI])/3.92. To estimate a pooled effect and corresponding 95% CI for the participants with OH at baseline versus those without, we weighted the logarithm of the hazard ratios by the inverse of their variance. The Cochrane’s Q test [15] and I^2^ test [18] were used to assess heterogeneity among studies. In the presence of relevant heterogeneity (I^2^>50%), we used random effect model to obtain a pooled estimate of effect. Sensitivity analyses [19] were conducted to evaluate the robustness of our results. We removed each study individually to evaluate that study’s effect on the summary estimates. Publication bias was evaluated by visually inspecting funnel plots for asymmetry [20]. We also performed the nonparametric “trim and fill” procedure [18] to further assess the possible effect of publication bias in our meta-analysis. This method considers the possibility of hypothetical “missing” studies that might exist, imputes their HRs, and recalculates a pooled HR that incorporates the hypothetical missing studies as though they actually existed.

## Results

### Search Results

**Figure 1:**
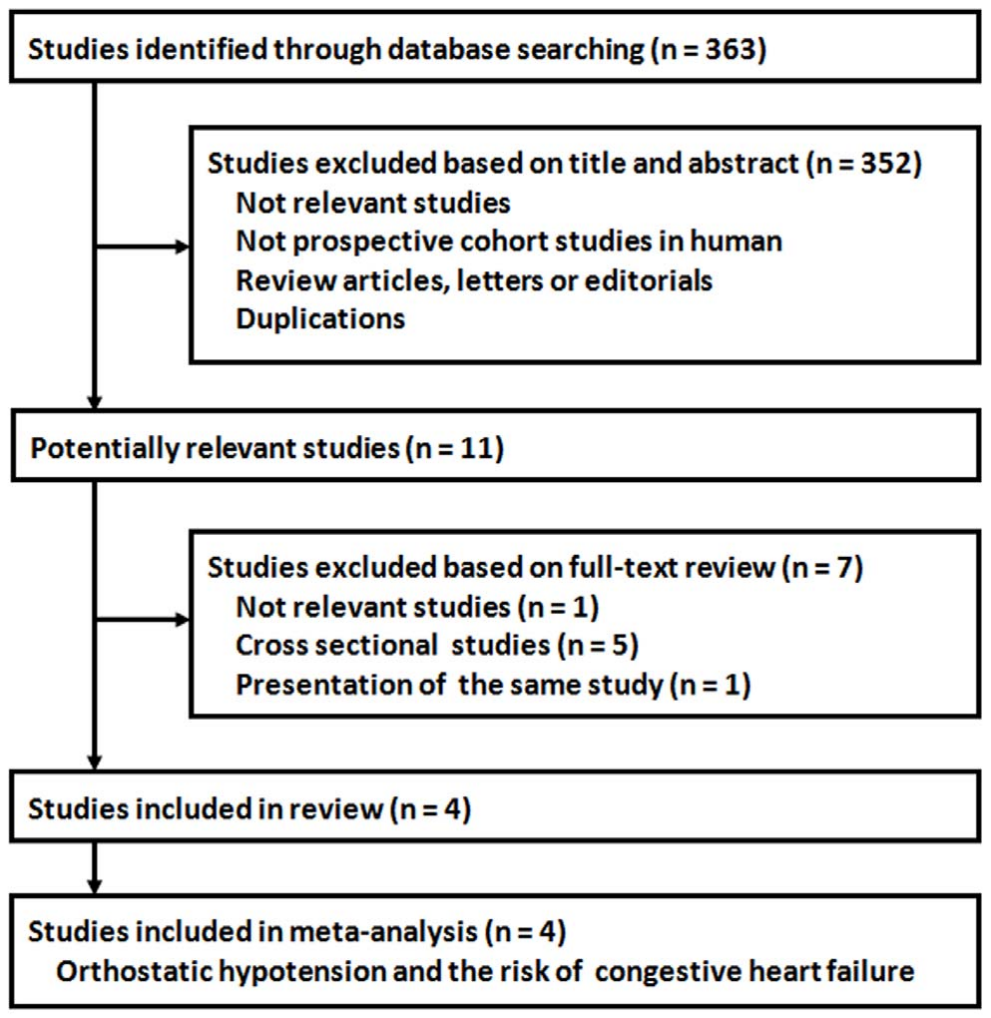
Search and selection of studies included in the meta-analysis.

The study selection process is shown in **Figure 1**. Overall, the database searching identified 363 citations from Pubmed and Embase, of which four studies [10]–[13] with 51270 participants and 3603 incident CHF cases were finally included in the meta-analysis.

### Study Characteristics

The characteristics of the included studies were shown in **Table 1** **and** **2**. Three of the studies [10]–[12] were conducted in Europe and North America, while the other one [13] was conducted in China and published in Chinese. All of the included studies were general population-based prospective cohort studies. The mean age of the participants in each study varied from 45.6 to 81.1 years, and the proportions of the male ranged from 38.4% to 95.6%. Two studies included a small proportion of patients with CAD at enrollment [11], [13], while didn’t for the other two studies [10], [12]. The definitions of OH in each study were consistent with the consensus of the international statement [16], although there were a little difference concerning the timing for measurements of SBP and DBP in standing position. The prevalence of OH at baseline varied between 5.0% and 25.6%. The studies generally defined incidence CHF as hospitalization for CHF or death from CHF. The mean follow-up duration varied from 1 year to 24 years, and the incidence of CHF outcomes varied from 1.7 to 17.1 per 1000 person-years in the studies. All of the included studies presented the association of OH and CHF incidence after fully adjusting the conventional cardiovascular risk factors, including age, gender, smoking status, BMI, prevalence of hypertension and DM.

**Table 1:** Baseline characteristics of prospective cohort studies included in the meta-analysis of orthostatic hypotension and incident heart failure.

Study	Country	Population	Baseline year	Ntotal	Male	Agerange	Meanage	BMI	HTN	DM	BaselineCAD	OH definition	OHprevalence
					%	years	years	kg/m^2^	%	%	%		%
**Verwoert** **2008**	the Netherlands	Community-living individuals	1990∼1993	5064	38.4	55∼99	68.1	26.2	26.9	8.9	0	SBP decline ≥20 mmHg or DBPdecline ≥10 mmHg from supineto standing (at 1, 2 or 3 minutes)	17.8
**Fedorowski** **2010**	Sweden	Population-basedmiddle-agedindividuals	1974∼1992	32669	68.2	26∼61	45.6	24.6	40.2	4.7	0.4	SBP decline ≥20 mmHg or DBPdecline ≥10 mmHg from supineto standing (within 3 minutes)	6.1
**Lin** **2011**	China	Elderly individualswho underwenthealth screening	2010	1174	95.6	65∼	81.1	25.1	72.3	41.2	7.8	SBP decline ≥20 mmHg or DBPdecline ≥10 mmHg from supineto standing (within 3 minutes)	25.6
**Jones** **2012**	USA	Community-livingmiddle-agedindividuals	1987∼1989	12363	45.3	45∼64	54.1	27.5	32.3	10.9	4.4	SBP decline ≥20 mmHg or DBPdecline ≥10 mmHg from supineto standing (within 2 minutes)	5.0

BMI, body mass index; HTN, hypertension; DM, diabetes mellitus; CAD, coronary artery disease; OH, orthostatic hypotension; SBP, systolic blood pressure; DBP, diastolic blood pressure.

**Table 2:** Congestive heart failure outcome definitions and follow up data in the prospective cohort studies included in the meta-analysis.

Study	CHF outcome definition	N total	Follow-up	N cases	Person-yearfollow-up	Event rates	Adjusted factors	Studyquality
			years			per 1000person-years		
**Verwoert** **2008**	Documented CHF (coded asI-50 for ICD-10, validatedusing European Heart failureguidelines) from GP, CHFhospitalization/death	5064	6.6	571	33422	17.1	Age, sex, smoking, use ofantihypertensive medications,BMI, SBP, DBP, DM, TCand HDL-C	9
**Fedorowski** **2010**	CHF hospitalization (coded as428 for ICD-9 or I-50 or I-11.0for ICD-10) documented bynational hospital dischargeregistry	32669	24.0	1293	784056	1.7	Age, sex, smoking, use ofantihypertensive medications,BMI, SBP, DBP, DM and TC	9
**Lin** **2011**	CHF hospitalization documentedby medical records anddischarge diagnosis byattending physicians	1174	1.0	19	1174	16.2	Age, sex, smoking, restingHR, BMI, SBP, DBP, DM,TC and prevalent CAD	7
**Jones** **2012**	CHF hospitalization/death(coded as 428 for ICD-9or I-50 for ICD-10)documented by hospitaldischarge registry or deathcertificate	12363	17.5	1720	216353	8.0	Age, sex, race, smoking, alcoholuse, educational level, restingHR, BMI, SBP, DM, HTN,LVH and prevalent CAD	9

Quality of the included studies were evaluated using the Newcastle-Ottawa Scale (range, 1∼9 stars). OH, orthostatic hypotension; CHF, congestive heart failure; GP, general practitioners; ICD, *International Classification of Diseases*; BMI, body mass index; SBP, systolic blood pressure; DBP, diastolic blood pressure; DM, diabetes mellitus; TC, total cholesterol; HDL-C, high density lipoprotein cholesterol; HR, heart rate; CAD, coronary artery disease; HTN, hypertension; LVH, left ventricular hypertrophy.

### Quality Assessment

The overall quality of studies included in the meta-analysis was good, with three studies [10]–[12] scoring 9 stars on the Newcastle-Ottawa scale and the other one [13] scoring 7 stars.

### Orthostatic Hypotension and the Risk of Congestive Heart Failure

**Figure 2:**
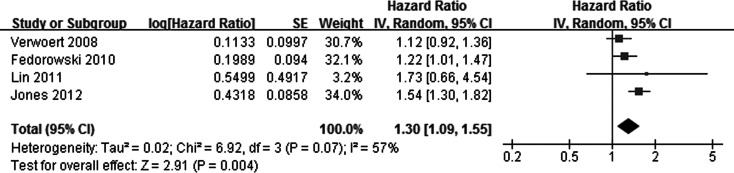
Adjusted hazard ratio of congestive heart failure in subjects with orthostatic hypotension at baseline compared those without orthostatic hypotension. CI, denotes confidence interval; the size of each square is proportional to the study’s weight (inverse of variance – IV).

All of the included studies showed that participants with OH at baseline had increased risk of CHF during follow-up compared with those without OH, although results of 2 studies [12], [13] were not significant. By pooling these studies together with a random effect model, result of our meta-analysis confirmed that participants with OH at baseline had a significant increased risk of incident CHF (adjusted HR: 1.30, 95% CI 1.09–1.55; p = 0.004, **Figure 2**). Significant heterogeneity was detected among the included studies (Cochrane’s Q test: p = 0.07, I^2^ = 57%). Sensitivity analysis by omitting one study at a time didn’t substantially change the overall result (adjusted HR 1.18 ∼ 1.39, p all <0.05; **Table 3**). In addition, we observed that no significant heterogeneity could be detected after the study by Jones et al. [11] was omitted (Cochrane’s Q test: p = 0.60, I^2^ = 0%).

**Table 3:** Sensitivity analysis.

Studies	p value for heterogeneity	I^2^	Pooled HR and 95% CI	p value for overall effect
**All included**	0.07	57%	1.30 [1.09, 1.55]	p = 0.004
**Exclude Verwoert 2008**	0.17	44%	1.39 [1.23, 1.57]	p<0.001
**Exclude Fedorowski 2010**	0.05	67%	1.35 [1.19, 1.53]	p<0.001
**Exclude Lin 2011**	0.04	70%	1.30 [1.17, 1.45]	p<0.001
**Exclude Jones 2012**	0.60	0%	1.18 [1.03, 1.35]	p = 0.01

HR, hazard ratio; CI, confidence interval.

### Influence of Participant Characteristics on the Association between Orthostatic Hypotension and the Risk of Congestive Heart Failure

**Figure 3:**
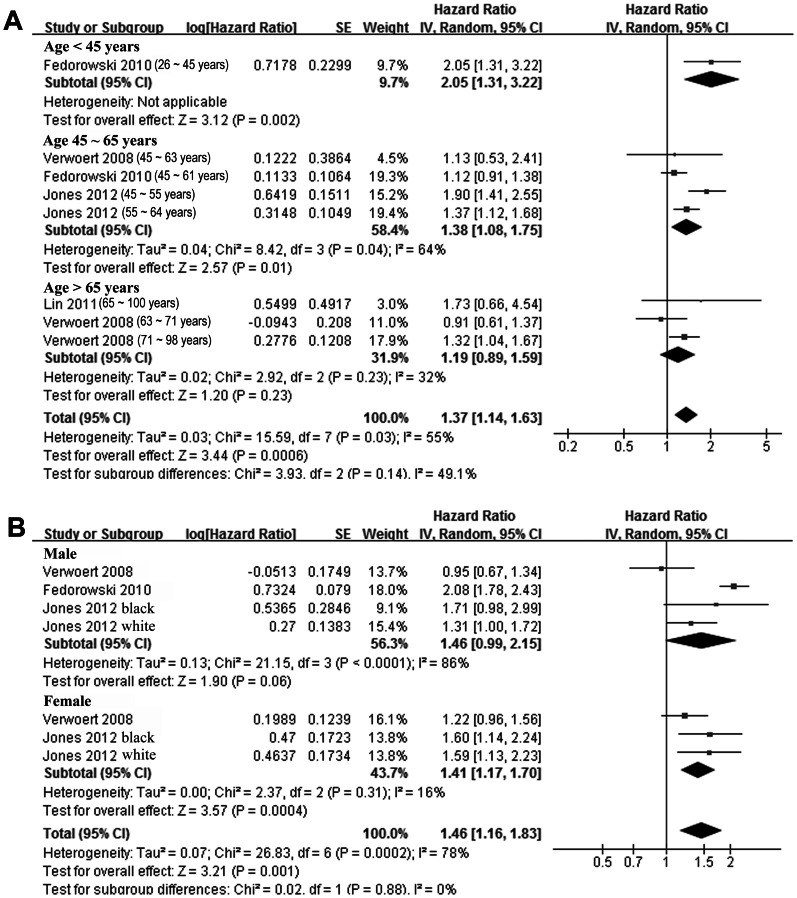
Adjusted hazard ratio of congestive heart failure in subjects with orthostatic hypotension at baseline compared those without orthostatic hypotension stratified by the age ranges (A) and gender (B) of the subjects. CI, denotes confidence interval; the size of each square is proportional to the study’s weight (inverse of variance – IV).

**Figure 4:**
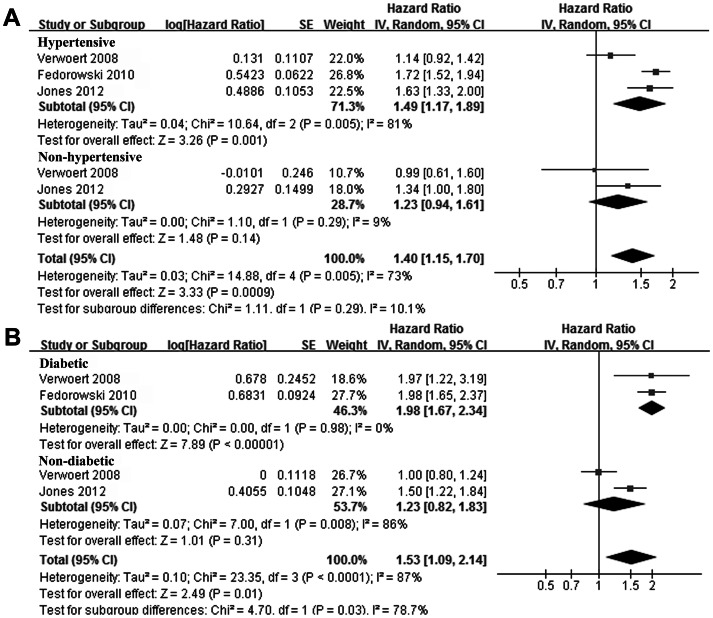
Adjusted hazard ratio of congestive heart failure in subjects with orthostatic hypotension at baseline compared those without orthostatic hypotension stratified by the comorbidities of hypertension (A) and diabetes mellitus (B) of the subjects. CI, denotes confidence interval; the size of each square is proportional to the study’s weight (inverse of variance – IV).

Since some of the studies [10]–[12] reported the association between OH and CHF risk according to the age range, sex, hypertension status and DM status of the participants, we combined these data to investigate whether these characteristics of the participants could affect the association between OH and CHF. Although the cut-off values of the age range in different studies were not exactly the same, by pooling of the data from similar age ranges, results of our stratified meta-analysis indicated that presence of OH seemed to increase the risk of CHF only in younger participants (<45 years or 45 ∼ 65 years), but didn’t in elderly participants (>65 years, **Figure 3A**). Presence OH increased the risk of CHF in both males and females (**Figure 3B**), which suggested that gender of the participants didn’t seem to affect the association between OH and CHF. In addition, we observed that presence of OH at baseline significantly increased the risk of CHF incidence in participants with hypertension or DM, but didn’t in those without these comorbidities (**Figure 4A and 4B**).

### Publication Bias

**Figure 5:**
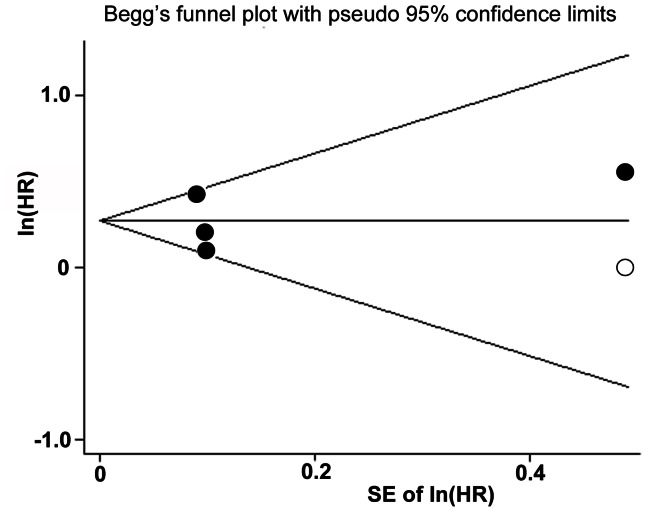
Funnel plots with trim and fill. The black dots present the identified studies included in the meta-analysis; the empty dot presents the estimated missing study after adjustment for publication bias.

The publication bias was difficult to describe because the number of studies included in the current meta-analysis is small, although the funnel plot was asymmetry by visually inspecting (**Figure 5**). We performed “trim-and-fill” analysis which conservatively imputes a hypothetical negative unpublished study in order to produce a symmetrical funnel plot. The pooled analysis incorporating the hypothetical study continued to show a statistically significant association between OH and CHF incidence (adjusted HR: 1.29, 95% CI 1.09–1.52, p = 0.002).

## Discussion

In this meta-analysis, by pooling the results of all available prospective cohort studies, we found that subjects with presence of OH were associated with about 30% higher risk of CHF incidence compared with those without OH at baseline. We also found that association between OH and increased CHF risk seemed to be significant in middle-age participants, and in those with hypertension or DM, but not significant in the elderly subjects, or in those without hypertension or DM. These results confirmed the hypothesis that individuals with OH, even without other CVD or risk factors, are at high risk for development of CHF in the future.

Although several previous cross-sectional studies have shown that presence of OH is independently related to left ventricular hypertrophy confirmed by electrocardiogram or echocardiography [21]–[23], the exact mechanisms underlying the significant predictive effect of OH for development of CHF are not known. Besides, at this stage, we can’t decide whether or not OH was causally related to CHF and whether or not OH was only a marker of a generally increased risk of CHF incidence. Several suggested mechanisms may be helpful to understand the relationship between presence of OH and increased risk for future CHF, of which autonomic dysfunction is an interesting one. It has been well recognized that baroreflex dysfunction probably induced by impairment of baroreceptor due to aging or atherosclerosis, is one of the most important causes of OH [6], [24], [25]. Indeed, when subjects stand up from a supine position, blunted baroreceptor may fail to deactivate the baroreflex and the vagal output therefore can not be down-regulated, leading to OH [6], [24]. On the other side, dysfunction of baroreflex has also been observed in CHF patients [26], [27]. Impaired baroreceptor may fail to activate the baroreflex in CHF patients with hemodynamic stress, and the sympathetic tone maintained activation, which has been accepted as an important mechanism underlying the pathogenesis and progression of CHF [26], [27]. Furthermore, increasing studies in both animal models [28] and human patients [29] suggested that activation of baroreflex with an implanted device may be a potential treatment for CHF, reflecting the fact that dysregulation of baroreflex may be an intermediate process for the association between OH and incident CHF. In addition to autonomic dysfunction, some other mechanisms have also been suggested to be involved, such as reduced coronary flow caused by frequent postural BP drop [30], increased early subclinical atherosclerotic burden [31], [32], abnormal nocturnal change in BP [33], and increased longstanding cardiovascular overload [22]. These mechanisms are also needed to be confirmed by future studies.

Our stratified analysis indicated that the significant association between OH and CHF incidence can be found in middle-age subjects and those with hypertension and DM at baseline. These results highlight the predictive effect of OH for future CHF in both the low-risk population and the high-risk population with known CHF risks. Although the results were not significant for elderly subjects and those without hypertension or DM, our study still found presence of OH increased the risk of future CHF in these subgroups (HRs all >1). In our point of view, these insignificant results may be attributed to the small number of the included studies.

Our study has some limitations which should be considered when interpreting the results. First, the number of studies included in the meta-analysis and stratifies analyses is small. The results for some subgroups should be interpreted with caution. Second, the CHF outcomes of the included studies were defined as CHF hospitalization or death. So, some less severe or asymptomatic CHF cases may not be included. Third, our meta-analysis is based on observational studies. Hence, we cannot exclude the chance, residual or unmeasured confounding. However, since there seemed to be no evidence-based effective intervention for the treatment of OH [34], it is difficult to confirm our results in a large randomized trial. Fourth, potential therapy to OH and many kinds of medications such as antihypertensive medication may affect the risk between OH and CHF. However, as indicated in a recent review [34], many commonly recommended interventions for OH have a limited evidence base supporting their use, and the effects have not been confirmed. Also, effects of some antihypertensive medications on OH, such as angiotensin-converting enzyme inhibitors, are not always consistent [6]. Furthermore, potential treatment for OH was not specified in the included studies, although the use of antihypertensive medications was adjusted in two of the studies when estimating the association between OH and incident CHF [10], [12]. We acknowledged that lack of controlling for potential treatment to OH and other medications is an important limitation of our study. Fifth, we did not have data on individual studies to assess CHF etiology (ischemic or non-ischemic) or types (preserved or reduced left ventricular systolic function). Nevertheless, our study also has numerous strengths, including the relative high quality of studies included (quality score ranging from 7 to 9), a large pooled sample size, robustness of the results in sensitivity analysis, and use trim-and-till analysis to handle potential publication bias.

In conclusion, results of our meta-analysis confirmed that presence of OH is related to a significant increased risk for development of CHF in the future, especially for the middle-age subjects and those with morbidities such as hypertension and DM. Studies are needed to explore the potential mechanisms underlying this association. More importantly, screen for OH may be of great clinical significance for the early identification of subjects at higher risk for development of CHF.
